# Axonal loss in major sensorimotor tracts is associated with impaired motor performance in minimally disabled multiple sclerosis patients

**DOI:** 10.1093/braincomms/fcab032

**Published:** 2021-03-16

**Authors:** Myrte Strik, L Eduardo Cofré Lizama, Camille J Shanahan, Anneke van der Walt, Frederique M C Boonstra, Rebecca Glarin, Trevor J Kilpatrick, Jeroen J G Geurts, Jon O Cleary, Menno M Schoonheim, Mary P Galea, Scott C Kolbe

**Affiliations:** 1 Department of Medicine and Radiology, University of Melbourne, Parkville 3010, Australia; 2 Department of Anatomy and Neurosciences, MS Center Amsterdam, Amsterdam Neuroscience, Amsterdam UMC, Vrije Universiteit Amsterdam, Amsterdam 1081 HZ, the Netherlands; 3 School of Allied Health, Human Services and Sports, La Trobe University, Victoria 3086, Australia; 4 Department of Neurosciences, Central Clinical School, Monash University, Melbourne 3004, Australia; 5 Florey Institute of Neuroscience and Mental Health, Parkville 3052, Australia; 6 Florey Department of Neuroscience and Mental Health, University of Melbourne, Parkville 3052, Australia; 7 Department of Neurology, Royal Melbourne Hospital, Parkville 3050, Australia; 8 Department of Radiology, Guy’s and St. Thomas’ NHS Foundation Trust, London SE1 7EH, UK

**Keywords:** multiple sclerosis, diffusion-weighted imaging, ultra-high field imaging, motor disability, axonal degeneration

## Abstract

Multiple sclerosis is a neuroinflammatory disease of the CNS that is associated with significant irreversible neuro-axonal loss, leading to permanent disability. There is thus an urgent need for *in vivo* markers of axonal loss for use in patient monitoring or as end-points for trials of neuroprotective agents. Advanced diffusion MRI can provide markers of diffuse loss of axonal fibre density or atrophy within specific white matter pathways. These markers can be interrogated in specific white matter tracts that underpin important functional domains such as sensorimotor function. This study aimed to evaluate advanced diffusion MRI markers of axonal loss within the major sensorimotor tracts of the brain, and to correlate the degree of axonal loss in these tracts to precise kinematic measures of hand and foot motor control and gait in minimally disabled people with multiple sclerosis. Twenty-eight patients (Expanded Disability Status Scale < 4, and Kurtzke Functional System Scores for pyramidal and cerebellar function ≤ 2) and 18 healthy subjects underwent ultra-high field 7 Tesla diffusion MRI for calculation of fibre-specific measures of axonal loss (fibre density, reflecting diffuse axonal loss and fibre cross-section reflecting tract atrophy) within three tracts: cortico-spinal tract, interhemispheric sensorimotor tract and cerebello-thalamic tracts. A visually guided force-matching task involving either the hand or foot was used to assess visuomotor control, and three-dimensional marker-based video tracking was used to assess gait. Fibre-specific axonal markers for each tract were compared between groups and correlated with visuomotor task performance (force error and lag) and gait parameters (stance, stride length, step width, single and double support) in patients. Patients displayed significant regional loss of fibre cross-section with minimal loss of fibre density in all tracts of interest compared to healthy subjects (family-wise error corrected *p*-value < 0.05), despite relatively few focal lesions within these tracts. In patients, reduced axonal fibre density and cross-section within the corticospinal tracts and interhemispheric sensorimotor tracts were associated with larger force tracking error and gait impairments (shorter stance, smaller step width and longer double support) (family-wise error corrected *p*-value < 0.05). In conclusion, significant gait and motor control impairments can be detected in minimally disabled people with multiple sclerosis that correlated with axonal loss in major sensorimotor pathways of the brain. Given that axonal loss is irreversible, the combined use of advanced imaging and kinematic markers could be used to identify patients at risk of more severe motor impairments as they emerge for more aggressive therapeutic interventions.

## Teaser

Multiple sclerosis is a neuroinflammatory disease of the CNS, associated with irreversible neuro-axonal loss, leading to permanent disability. Strik et al. report that advanced diffusion MRI reveals diffuse axonal loss in major sensorimotor pathways that correlate with subtle impairments in lower limb motor control and gait in minimally disabled patients.

## Graphical Abstract

**Graphical Abstract:**
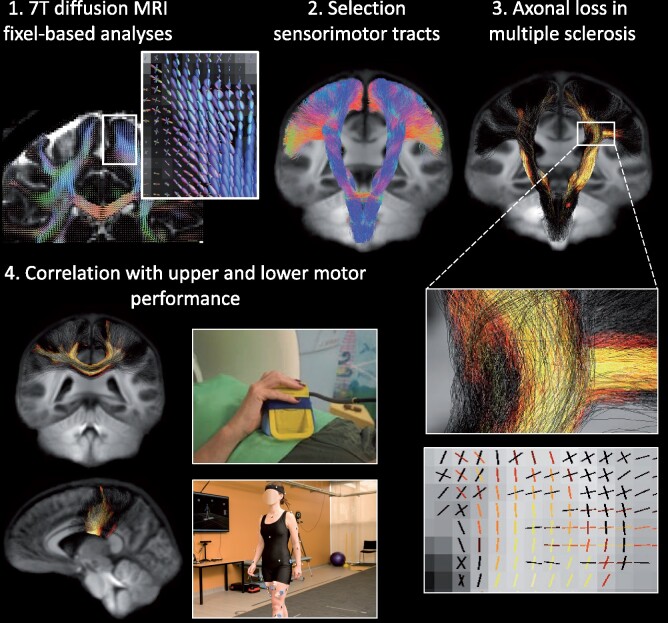

## Introduction

Multiple sclerosis (MS) is an autoimmune-mediated disorder of the central nervous system, common in young adults. Over the longer term, many patients develop progressively worsening walking and dexterity impairments that ultimately affect performance of daily-life activities substantially.^1^ Progression of motor disabilities is associated axonal loss in clinically eloquent white matter (WM) pathways such as the corticospinal tract (CST),^2^ with significant axonal loss occurring prior to clinical progression. Thus, identifying patients at greatest risk of progressive sensorimotor decline requires both *in vivo* markers for axonal loss and sensitive functional markers that can detect subtle impairments and walking and hand function that predate overt clinical impairment.

Axonal loss can be detected *in vivo* using diffusion MRI that is sensitive to the microscale motion of water molecules in tissue that are restricted in healthy WM due to the multitudinous collinear membranes. In WM structures containing a single fibre population, diffusion can be adequately modelled using a tensor ellipsoid (diffusion tensor imaging, DTI). Previous studies have reported loss of diffusion anisotropy in important sensorimotor pathways such as the CST,^2–4^ corpus callosum^5^^,^^6^ and thalamus,^7^^,^^8^ and DTI metrics have been shown to more strongly correlate with neurological disabilities than conventional MRI measures such as lesion load.^9^ Recently, it has become recognized that DTI is insufficient to model axonal loss due to: (i) sensitivity to crossing or merging fibres that include > 90% of brain voxels^10^ and (ii) sensitivity to non-axonal cellular pathologies such as increased extracellular fluid and non-neuronal cells such as inflammatory cells.^11^ These limitations have led to the development of more advanced diffusion MRI acquisition and signal modelling techniques to allow estimation of tract-specific axonal fibre density (FD) and fibre cross-section using fixel-based analysis.^12–15^ In MS, fixel-based analysis has been shown to more sensitively and specifically identify microstructural changes in damaged WM compared to DTI.^16^^,^^17^ Moreover, using the high signal-to-noise ratio afforded by ultra-high field (7 Tesla) MRI and simultaneous multi-slice imaging techniques, diffusion MRI can be acquired with spatial resolution approaching anatomical imaging and high angular resolution within reasonable scan times (∼10 min).

Kinematic analyses of video or force tracking can reveal subtle changes in upper and lower limb motor control before clinical impairments become overt.^18^^,^^19^ Previous studies have shown that DTI changes within the CST are associated with impairments in simple gait assessments such as timed walks, strength or up and go tests.^3^^,^^20^^,^^21^ Reduced strength^20^ and slower walking speed were associated with reduced CST fractional anisotropy^20^ and increased radial and axial diffusivity.^3^^,^^21^ These walking metrics explained more variance in CST diffusivity than basic clinical measures such as Expanded Disability Status Scale (EDSS), age and gender.^20^ Another study did not find a relation between timed up and go and walking with DTI metrics, but reported a link with grey matter (GM) volume loss.^22^ However, there has been limited work comparing modern and more sensitive kinematic assessments such as 3D motion or force tracking with sensorimotor tract damage,^3^ particularly in minimally disabled patients. So, it is not clear whether the additional sensitivity to change of these new techniques relates to physiological (potentially modifiable through physical or symptomatic treatment) or structural (irreversible but can be delayed through more aggressive disease-modifying treatment) brain changes. Sensitive tracking of both functional impairments and associated brain changes is therefore important for assisting treating clinicians to better manage the progression of the disease.

This study therefore aimed to examine relationships between advanced diffusion MRI markers for axonal loss in major sensorimotor pathways of the brain and kinematic analyses of gait and upper/lower limb motor control in people with MS and minimal clinical disability. We hypothesized that patients would display subtle impairments in gait and motor control that would be associated with axonal loss in major sensorimotor tracts.

## Materials and methods

### Participants

This study was conducted in accordance with the Declaration of Helsinki with the protocol and informed consent forms approved by the Human Research Ethics Committees of the Royal Melbourne Hospital. All participants provided voluntary, written consent to one of the investigators. We recruited 28 MS patients (age = 41.9 ± 10.0 years; 23 females) from the Royal Melbourne Hospital, Melbourne, Australia, and 18 healthy controls (HC) (age = 39.2 ± 7.1 years; 10 females) through word-of-mouth advertising. At the time of assessment, all patients were diagnosed with clinically definite MS according to the revised criteria of McDonald.^23^ All patients presented with minimal disability (EDSS < 4, and Kurtzke Functional Scale for pyramidal and cerebellar function ≤ 2). No patients performed physical rehabilitation and or specific physical activities before the study enrolment. Patients were excluded with a disease duration of more than 20 years, evidence of a clinical relapse in the 6 months prior, neurological conditions other than MS, orthopaedic conditions causing disability of the lower limbs, or cardiovascular diseases.

### Upper and lower limb tracking task

Two behavioural task parameters were computed from data acquired during a functional MRI (fMRI) acquisition performed during the same MRI acquisition session (Supplementary Fig. 1) with methods related to these tasks reported elsewhere.^24^ To briefly reiterate, participants performed two runs of a visually guided force-matching task that involved a controlled, low force contraction of either (i) the ankle (the tibialis anterior) or (ii) hand muscles. The lag and force error of the upper and lower limb tasks was calculated from the force data collected during testing. Lag was the delay between the force target stimulus and the actual force response, calculated using the maximum cross-correlation between stimulus and response time series (milliseconds). The force error, a measure of accuracy and sensorimotor integration error, was the root mean square difference between the stimulus and the lag-corrected response time series (Newtons).

### Spatial-temporal gait assessments

Gait assessments were performed on the same day as MRI (*n* = 9), or within a median time of 14.5 days, (IQR = 0.75 - 151.50) from the MRI scan (*n* = 19). For gait assessments, reflective markers were placed on participant’s specific body landmarks following the full-body plug-in-gait model (VICON, Oxford, UK) (Supplementary Fig. 1). We asked participants to walk barefoot on a 12 m long walkway at 1.4 ms^−1^ with a tolerance of ± 5% of the target speed, which was monitored using two timing gates separated by 3 m in the middle of the walkway. Participants were requested to modify walking speed, if necessary, by providing verbal feedback on the speed recorded. All walking trials were recorded until five left and five right gait cycles at each speed were collected. Marker kinematics was collected using an 8-MX VICON camera system and Nexus 2 software (VICON, Oxford, UK). Raw data were exported and analysed using a customized Matlab R2020a (Mathworks, Natick, MA, USA) script to calculate basic spatiotemporal measures of gait based on feet markers (heel, ankle and toe) including: stride length; step width; and time percentage spent in single and double support and stance gait phases. Markers at the sacrum level were used to calculate overall gait speed. For each participant, spatiotemporal measures obtained from five left and five right gait cycles at each speed were averaged for further statistical analyses.

### Imaging acquisition

All participants were imaged using a whole-body Siemens MAGNETOM 7 Tesla MRI system (Siemens Healthcare, Erlangen, Germany) with a combined single-channel transmit and 32-channel receive head coil (Nova Medical, Wilmington, MA, USA). High-resolution diffusion-weighted imaging (DWI) was acquired using a simultaneous multi-slice 2D spin-echo echo-planar imaging (EPI) sequence based on a published sequence^25^ with the following parameters: repetition time (TR) = 7000 ms, echo time (TE) = 72.4 ms, flip angle (FA) = 90/180 degrees, multiband factor (MB) = 2, GRAPPA phase acceleration factor = 3, 128 slices, phase encoding direction (PE) anterior-to-posterior (AP), 1.24 mm isotropic resolution, whole-brain coverage and 103 directions. DWI imaging was acquired using multiple b-shells including 5 b = 0 s/mm^2^, 17 b = 1000 s/mm^2^, 31 b = 2000 s/mm^2^ and 50 b = 3000 s/mm^2^ images. In addition, six non-diffusion-weighted images (b = 0 s/mm^2^) with opposite phase encoding (posterior-to-anterior) were acquired to correct for susceptibility-induced off-resonance field distortion.

Two runs of fMRI were acquired (upper and lower limb motor tasks performed in separate runs) with the following parameters: TR = 1700 ms, TE = 34.4 ms, FA = 65 degrees, MB = 6, GRAPPA phase acceleration factor = 2, 120 slices, 1.2 mm isotropic resolution, 165 volumes, image matrix = 168 × 168.

A 3D T_1_-weighted structural scan [MP2RAGE: TR = 4900 ms, TE = 2.89 ms, inversion time (TI) = 700/2700 ms, flip angle (FA) = 5/6 degrees, 192 slices, AF = 4, Phase Encoding direction = AP, voxel size = 0.9 mm isotropic, image matrix = 256 × 256] and 2D fluid-attenuated inversion recovery scan (FLAIR) (TR = 10000 ms, TE =96 ms, TI = 2600 ms, flip angle = 145 degrees, 45 axial slices, GRAPPA = 3, PE = AP, voxel size = 1.2 × 1.2 × 3.0 mm) were acquired for volumetric measurements and lesion identification.

All patients were also imaged using a Siemens Prisma 3 Tesla MRI system (Siemens Healthcare, Erlangen, Germany) using a 64 channel combined head neck receive coil with body transmit for collection of a sagittally acquired 3D fluid-attenuated inversion recovery (FLAIR) scan for spinal cord lesion identification [TR = 5000 ms, TE = 408 ms, inversion time (TI) = 1800 ms, flip angle (FA) = 120 degrees, 160 slices, AF = 4, phase encoding direction = AP, voxel size = 0.98 mm isotropic, image matrix 256 × 256].

### Anatomical MRI processing

WM lesions were segmented on the MP2RAGE using a semi-automated thresholding technique in MRIcron (https://www.nitrc.org/projects/mricron). To avoid inclusion of cerebrospinal fluid, the 7T FLAIR image was used as visual reference. To create a lesion probability map, individual lesion maps were warped to the diffusion group template using Advanced Neuroimaging Tools (ANTs, v.2.3.1, http://stnava.github.io/ANTs/) antsRegistrationSyNQuick and antsApplyTransforms scripts, binarized and merged. In addition, the percentage intersection between the probability map and tracts of interest was calculated. MP2RAGE images were lesion filled with lesion maps using SLF (http://atc.udg.edu/nic/slfToolbox/index.html) in Statistical Parametric Mapping (SPM8), which were subsequently used for brain segmentation analyses using FreeSurfer version 6.0-patch (https://surfer.nmr.mgh.harvard.edu/). FreeSurfer volumetrics used in statistical analysis included the WM, cortical GM, deep GM, ventricular volumes and right and left thalamus, cerebellum, pre-central gyrus and post-central gyrus. All volumetrics were normalized by total intracranial volume. One subject’s data could not be reliably segmented, even after lesion filling, and were excluded from volumetric analyses.

Spinal cord cross-sectional area was measured on MP2RAGE scans at the level of the superior margin of the odontoid peg (Supplementary Fig. 2). Sagittal images were used to reconstruct transverse images perpendicular to the cord and the cord was manually marked using Horos (v4.0, horosproject.org). While no dedicated spinal cord imaging was performed in this study, cervical spinal cord lesions were manually segmented up till cervical cord area 4 using 3T FLAIR scans.

### Diffusion-weighted MRI analyses

**Figure 1:**
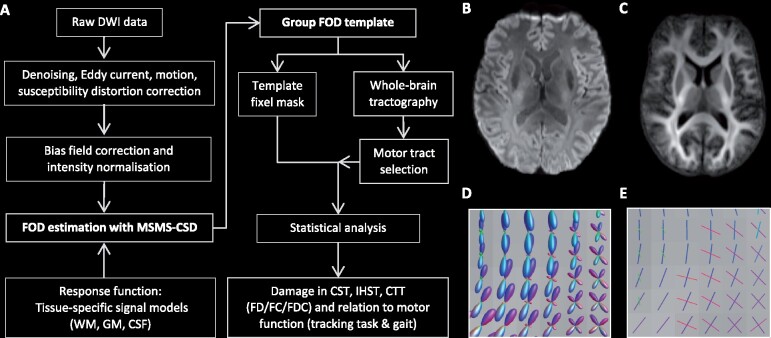
**Overview processing pipeline.** **(A, B)** The diffusion weighted imaging (DWI) processing pipeline. **(B)** DWI after pre-processing steps. **(C)** The WM FOD group template. **(D)** WM FODs colour coded by direction (green: dorsal-ventral, red: left-right, blue: cranial-caudal) and **(E)** corresponding fixels. CST = corticospinal tracts; CTT = cerebello-thalamic tracts; FC = fibre cross-section; FD = fibre density; FDC = fibre density and cross-section; GM = grey matter; IHST = interhemispheric sensorimotor tracts; MSMT-CSD = multi-shell multi-tissue constrained spherical deconvolution.

Diffusion imaging analyses were performed with MRtrix3, using the multi-shell multi-tissue constrained spherical deconvolution method (Fig. 1). Having multiple b-shells allowed us to differentiate between macroscopic tissue types including the WM, GM and cerebrospinal fluid resulting in a more complete modelling of the diffusion-weighted signal and more precise estimation of the fibre orientation distribution (FOD) and accurate measures of axonal damage.^26^

### Pre-processing diffusion-weighted imaging data

Diffusion-weighted MRI pre-processing involved denoising, motion and distortion correction including eddy current-induced distortions (EDDY, FSL, v.6.0.3) and susceptibility-induced EPI distortions using the anterior-posterior phase-encoded non-diffusion-weighted images (TOPUP, FSL, v.6.0.3). One subject did not have reverse phase-encoded data correctly acquired and therefore was not processed for EPI distortion correction. Next, images were corrected for bias field signal (ANTs, N4 algorithm, http://stnava.github.io/ANTs/) and global intensity normalization was performed across all participants using the median b = 0 s/mm^2^ intensity within a WM mask for robust voxel-wise comparisons across participants and to correct for residual intensity inhomogeneities.^13^

### Fibre orientation distribution estimation

To accurately and specifically estimate the FOD of the WM, multi-shell multi-tissue constrained spherical deconvolution was applied using tissue-specific response functions for WM, GM and cerebrospinal fluid by exploiting the unique b-value dependencies of each tissue type.^26^ The response function represents the diffusion signal of in a pure voxel of WM (single fibre), GM or cerebrospinal fluid, and is then used to estimate the FOD for each tissue class using constrained spherical deconvolution of the diffusion-weighted signal. The estimation of the response function and distribution of fibres was fully automated and driven by the DWI data itself. While we focused solely on the WM FODs as measure WM tissue damage, the GM and cerebrospinal fluid tissue classes in the model account for the remainder of the complete diffusion-weighted signal making the WM FOD estimation more accurate. In addition, modelling three-tissue FODs results in more precise FOD estimates at tissue boundaries what results in more accurate measures of axonal damage and fibre tracking.^26^

### Group template construction

A study-specific group WM FOD template was created using a randomly chosen subset of participants (10 MS patients and 10 HC) by repeatedly registering (non-linear) the WM FOD map of each subject to a continually updated average template image (Fig. 1). The WM group template was used in all subsequent steps including calculations of fibre-specific measures of axonal damage and statistical analysis. For each subject, the FOD images were registered to the group WM FOD template generating a deformation field that encodes non-linear transformations. To ensure subsequent analysis steps are performed in voxels with data in all participants, an intersection mask is created and used to segment fixels, individual fibre populations, from the FOD template, i.e. the fixel template analysis mask. Each subject’s WM FOD image was non-linearly aligned to the WM FOD group template and correspondence between each subject’s fixel map and the template fixel mask was identified (Fig. 1). For each fixel, three measures were calculated: FD, fibre cross-section (FC) and the FD modulated by cross-section (FDC which equals FD × FC).

### Identification of tracts of interest

Firstly, whole-brain probabilistic fibre tractography was performed on the group WM FOD template using the following stopping criteria: minimum angle = 22.5, maximum length = 250 mm, minimum length = 10 mm, power = 1.0, 20 million seeds randomly assigned within the WM template mask (Video 1). To improve on the reconstruction of the structurally connected fixels and to reduce biases in tractogram densities, the tractogram was filtered to 2 million tracts using the SIFT2 algorithm.^27^ Subsequently, the following tracts were selected using inclusion and exclusion regions of interest:

#### Corticospinal tracts

**Figure 2:**
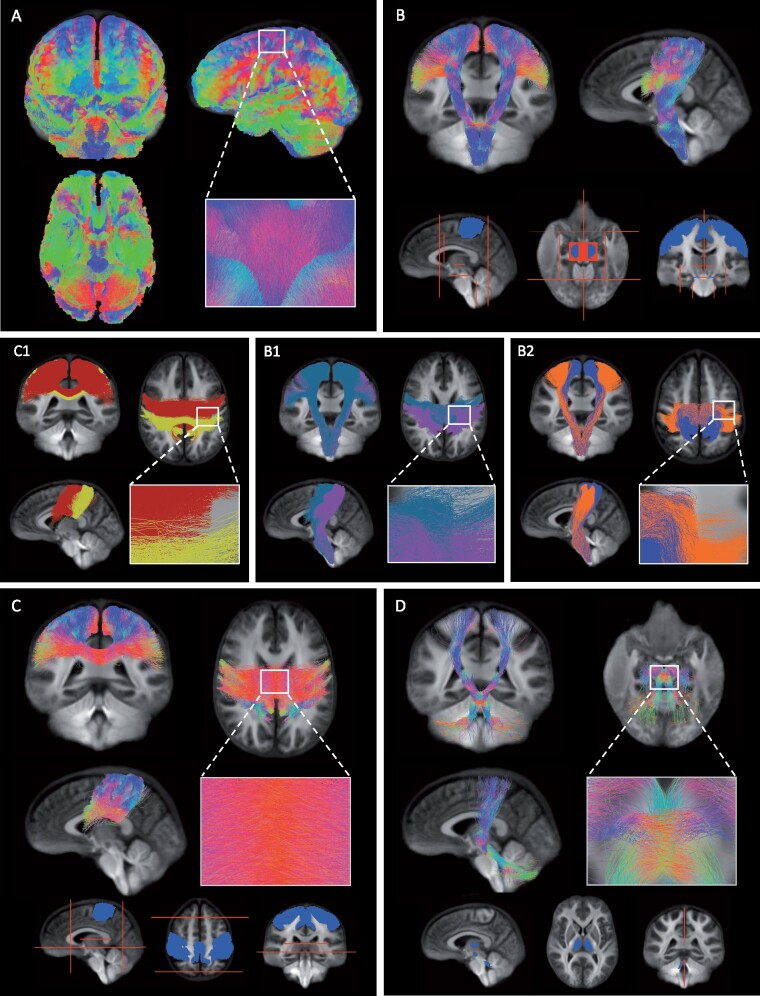
**The tracts of interest.** **(A)** Whole-brain tractogram was created on the group WM fibre orientation template using probabilistic tractography. **(B)** The CST was identified from the whole-brain tractogram using seed regions of interest located in the primary motor cortex (M1), primary somatosensory cortex (S1) and midbrain (blue). Exclusion regions were drawn manually to exclude tracts entering the cerebellum, thalamus and corpus callosum, and tracts terminating posterior or anterior to the CST (red). Within the CST, we investigated the proportion of damage observed within **(B1)** M1 (blue) and S1 (pink) tracts using either the pre-central or post-central gyrus as inclusion region and **(B2)** tracts related to upper (blue) or lower limb (orange) movements specifically using fMRI activation maps. **(C)** The IHST were selected using the right and left pre- and post-central gyrus (blue) and to prevent tracts from running anterior, posterior or inferior several exclusion planes were used (red). **(C1)** The interhemispheric tracts between left and right S1 (yellow) and left and right M1 (red). **(D)** The CTT were selected using seed regions within the superior cerebellar peduncle, red nucleus and thalamus (blue) and two sagittal exclusion planes were used superior and inferior of the decussation (red). **(A–D)** The colour of the tracts indicates the local fibre orientation (green: dorsal-ventral, red: left-right, blue: cranial-caudal).

The regions used to select the CST from the whole-brain tractogram included pre-central (M1) and post-central (S1) gyrus GM masks included in the Harvard Oxford Atlas (provided by FSL, FMRIB, Oxford, UK), which were registered to the diffusion group template using ANTs (ANTs, v.2.3.1, http://stnava.github.io/ANTs/), thresholded at 30% (voxels that were in 30% of subjects for those ROIs) and combined to form one inclusion region (Fig. 2, Video 2). In addition, two midbrain masks were manually drawn and used as inclusion regions. Regions of interest were manually drawn to exclude tracts entering the corpus callosum, cerebellum and thalamus, and tracts terminating anterior or posterior to the CST.

The CST tract was further subdivided into M1 and S1 tracts (Fig. 2) and tracts related to upper and lower limb movements (Fig. 2) using the follow method:

- M1 and S1 tracts were selected using the M1 as inclusion region for M1 fibres and as exclusion regions for S1 tracts and vice versa.
- The upper limb and lower limb tracts within the CST were selected using the fMRI task data.^24^ Patients performed two visually guided force-matching tasks involving a low force contraction of the lower limb (tibialis anterior) or upper limb (hand muscles). First, functional activation patterns specific to upper or lower limb movement were estimated using a two-level analysis (run and higher-level) using FEAT (FSL, FMRIB, Oxford, UK, v.6.0.3). Significant voxels were identified using family-wise error correction (threshold of z-stat > 2.3 and cluster size significance *p* < 0.05). Run-level activation maps from all participants were used as input to higher-level analyses to calculate activation maps specific to either upper or lower limb movements (upper > lower and lower > upper). Significant upper and lower activation maps were registered to the diffusion group template using ANTS and subsequently used as inclusion regions to select tracts specific to either upper or lower limb movements within the CST.

#### Interhemispheric sensorimotor tracts

The interhemispheric sensorimotor tracts (IHST) were selected using the left and right M1 and S1 as inclusion regions (Fig. 2). To exclude tracts running anterior or posterior, two coronal planes were used and an axial plane to prevent tracts running inferior. The IHST was further subdivided into M1 and S1 interhemispheric tracts (Fig. 2, Video 3). These tracts were selected using the right and left M1 as inclusion regions for M1 interhemispheric connections and as exclusion regions for the S1 interhemispheric tracts and vice versa.

#### Cerebello-thalamic tracts

To select the cerebello-thalamic tracts (CTT), we used the 20 million tract tractogram instead of the SIFT filtered tractogram to increase the number of tracts available. We manually created regions of interest for the left superior cerebellar peduncle, right red nucleus and right thalamus as well as right superior cerebellar peduncle, left red nucleus and left thalamus. Two sagittal planes were used to prevent tract running across hemispheric, just inferior and superior of where the CTT tracts decussate (Fig. 2, Video 4). We combined both tracts to form one tract of interest and tracts running from the thalamus to the cortex were not included in the statistical analyses.

### Statistical analysis

Groups were compared on demographics, brain volumetrics, spatiotemporal gait and functional behavioural parameters using independent samples *t*-tests, or when data were non-normally distributed, Mann–Whitney U-tests and Chi-square test for nominal variables. Five additional healthy control participants with gait analysis but no MRI data available were included in group comparisons involving gait parameters to improve statistical power.

Fixel-wise general linear models with connectivity-based fixel enhancement (*p*_fwe_ < 0.05) and non-parametric permutation testing (*n* = 5000 samples)^12^ were used to compare FD, FC and FDC within the CST, IHST and CTT tracts between MS patients and HC, and for correlations between fixel metrics and spatial-temporal gait measures (stance, stride length, step width, single and double support) and behavioural parameters (lag and force error).

*Post hoc* analyses were performed to calculate the proportional volume of M1, S1, upper limb and lower limb tracts within the CST and M1 and S1 interhemispheric tracts within IHST that displayed significant group differences. In addition, linear regression analyses were performed between the mean significant fixel metrics within damaged tracts and EDSS, and volumetrics in MS patients. Residuals were tested for normality and partial rank correlations were performed when non-normally distributed. For all regression analyses, age and sex were included as covariates, significance was set at *p *<* *0.05. Corrections for multiple comparisons were performed using false discovery rate (FDR).^28^

### Data availability

Deidentified data for this study are available upon reasonable request to the corresponding author.

## Results

### Demographics, volumetric measures and clinical disability

Demographics, volumetric measures and clinical disability scores (EDSS) are summarized in Table 1. MS patients did not significantly differ in sex, age or hand dominance from HC.

**Table 1:** Demographics, clinical and MRI characteristics

	HC (*n* = 18)	MS patients (*n* = 28)	*p* **-value**	*p* **-value FDR**
**Demographics**				
Sex, F/M^b^	10/8	23/5	0.051	0.108
Age	39.22 (7.13)	41.75 (10.01)	0.358	0.380
Disease duration		6.50 (3.94)		
Dominant hand (R/L), *n*^b^	15/3	27/1	0.124	0.211
**Disability scores**				
EDSS^a^		1.50 (1.0, 1.5)		
Pyramidal FSS^a^		1.00 (0.0, 1.0)		
Cerebellar FSS^a^		0.00 (0.0, 1.0)		
**Brain volumetrics**				
BP, mm^3^	1 143 921 (100 661)	1 061 714 (90 033)	0.002^*^	0.007^*^
WM, mm^3^	423 592 (47 671)	380 951 (48 597)	0.001^*^	0.006^*^
CGM, mm^3^	399 123 (36 891)	377 639 (32 317)	0.007^*^	0.020^*^
DGM, mm^3^	75 246 (8571)	67 692 (7090)	0.002^*^	0.009^*^
Ventricles, mm^3^	26 334 (18 082)	34 350 (17 808)	0.106	0.200
M1 L, mm^3^	13 515 (1760)	12 725 (2636)	0.196	0.278
M1 R, mm^3^	13 034 (1852)	12 444 (2814)	0.336	0.381
S1 L, mm^3^	9843 (1202)	9013 (1954)	0.018^*^	0.044^*^
S1 R, mm^3^	9339 (1046)	8870 (2024)	0.195	0.301
Cerebellum L, mm^3^	49 466 (4570)	46 611 (9783)	0.231	0.280
Cerebellum R, mm^3^	49 639 (4496)	46 745 (10 128)	0.218	0.285
Thalamus L, mm^3^	6962 (707)	5832 (1190)	<0.001^*^	<0.001^*^
Thalamus R, mm^3^	6673 (625)	5695 (1113)	<0.001^*^	<0.001^*^
Lesion volume, mm^3^		3303 (5248)		
Spinal cord lesion volume, mm^3^		176 (248)		
Spinal cord, mm^2^	69.55 (6.64)	71 (10)	0.703	0.703

Compared to healthy controls (HC), multiple sclerosis (MS) patients displayed significantly reduced brain parenchymal (BP), WM, cortical grey matter (CGM), deep grey matter (DGM), right and left thalamus and left primary somatosensory cortex (S1) volume. Besides lesion load and spinal cord area, all volumetrics were normalized for intracranial volume. One subject was excluded from volumetric analyses as we were unable to reliable to calculate volumetrics even after lesion filling. All variables were tested using independent samples *t*-test and values represent means and standard deviations unless denotes otherwise.

a Median and interquartile range.

b Chi-square test.

* Significant difference between MS patients and HC.

F = female; M = male; R = right; L = left; EDSS = Expanded Disability Status Scale; M1 = primary motor cortex.

MS patients displayed significant reduction in whole-brain volumetrics including cortical GM (*p*_FDR_ = 0.020), deep GM (*p*_FDR_ = 0.009) and WM volume (*p*_FDR_ = 0.006), compared to controls. In addition, MS patients showed significant right (*p*_FDR_ < 0.001) and left (*p*_FDR_ < 0.001) thalamic atrophy and reduced volume of the left post-central gyrus (S1) (*p*_FDR_ = 0.044), compared to controls.

MS participants displayed greater force error (*p*_FDR_ = 0.010) and a longer lag (*p*_FDR_ = 0.010) during lower limb force matching but not during upper limb movements (Table 2). MS patients walked with a shorter stride length compared to HC, but the *p*-value did not survive FDR correction (*p *=* *0.021, *p*_FDR_ = 0.070) (Table 2).

**Table 2:** Functional motor performance and spatiotemporal gait measures

	HC	MS patients	*p* **-value**	*p* **-value FDR**
**Force-matching performance**				
Number of participants	17	28		
Upper limb lag, ms	184.71 (113.75)	216.07 (90.08)	0.201	0.503
Upper limb force error, *N*	0.31 (0.07)	0.34 (0.09)	0.226	0.452
Lower limb lag, ms	142.35 (116.49)	266.43 (120.68)	0.002*	0.010*
Lower limb force error, *N*	0.30 (0.05)	0.45 (0.16)	0.001*	0.010*
**Spatiotemporal gait measures**				
Number of participants	15	28		
Speed, ms	1.39 (0.04)	1.38 (0.03)	0.308	0.513
Single support, % gait cycle	40.19 (3.26)	40.45 (2.92)	0.760	1.000
Terminal double support, % gait cycle	7.79 (1.30)	7.78 (1.54)	0.985	0.985
Stance, % gait cycle	58.52 (1.26)	58.44 (2.35)	0.899	1.000
Stride length, mm	1426.26 (88.09)	1358.05 (89.16)	0.021*	0.070
Step width, mm	75.51 (22.68)	77.26 (24.50)	0.919	1.000

Compared to HC, MS patients showed worse behavioural performance during the lower limb visually guided motor task during fMRI testing, but no differences were observed during upper limb movement. MS patients displayed longer lag *(p *=* *0.002) and more force error (*p *=* *0.001) during lower limb movements, compared to HC. In addition, during walking patients showed a shorter stride length compared to controls *(p*=* *0.021). *Significant difference between MS patients and HC.

### Sensorimotor network damage and conventional MRI

**Figure 3:**
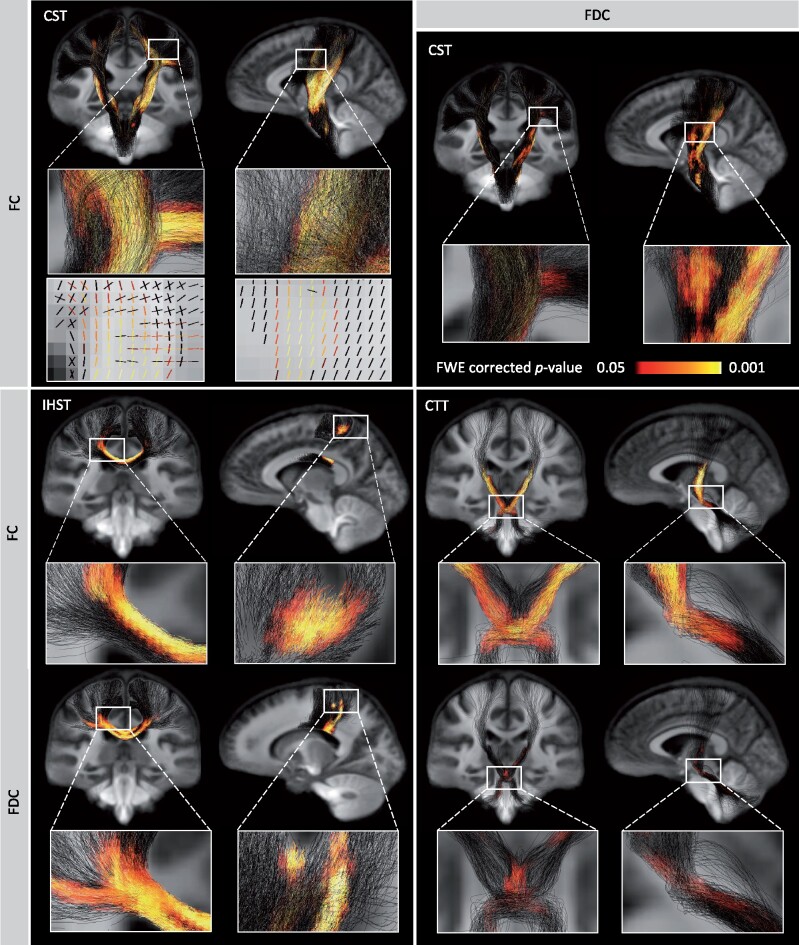
**Damage within the tracts of interest.** MS patients showed substantial loss of fibre cross-section (FC) and to a lesser extent loss of fibre density and cross-section (FDC) in the corticospinal tracts (CST), IHST and cerebellar-thalamic tracts (CTT), compared to HC. Significant fixels [family-wise error (FWE) corrected *p*-value < 0.05] are represented on the tractogram and coloured with the *p*-value and a close-up of the significant fixels are displayed for FC in the upper panel.

For the CST, FC and FDC were significantly reduced in MS patients in large regions of the tracts with no difference in FD compared to HC (Fig. 3, Video 5). Within the CST, we assessed the proportional volume of M1, S1, upper limb and lower limb connected tracts that overlapped with significant group differences. The largest volume of significant difference was observed for FC. This region overlapped with 20.2% of M1 tracts, 27.3% of S1 tracts, 35.7% of lower limb tracts and 29.7% upper limb tracts. Areas with reduced FDC overlapped with 6.8% of M1 tracts, 11.6% of S1 tracts, 14.2% of lower limb tracts and 12.4% upper limb tracts.

For the IHST, MS patients showed significantly reduced values for all fixel-specific metrics compared to HC, mostly within the corpus collosum (Fig. 3). The strongest significant differences were observed for FC (Video 6) and this region intersected with 4.6% of M1 interhemispheric tracts and 10.9% of S1 interhemispheric tracts. More significant fixels were observed for FDC, which overlapped with 2.6% of M1 and 13.2% of S1 interhemispheric tracts. Reduction in FD was observed to a much smaller extent and overlapped with only 0.02% of M1 and 0.38% of S1 interhemispheric tracts.

For the CTT, patients showed significantly reduced FC (Video 7) and to a lesser extent FDC, but not for FD, compared to controls (Fig. 3).

**Figure 4:**
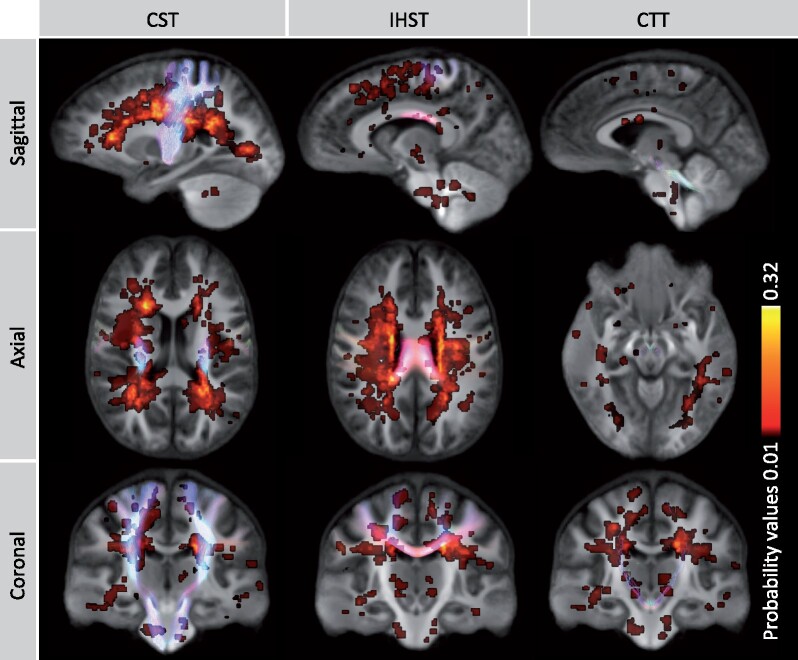
**Lesion probability map and tracts of interest.** This figure demonstrates the probabilities of lesions occurring across the brain across MS patients with the motor tracts visualized in an overlying manner. Low probability values are shown in red and higher values are shown in yellow. The maximum probability was 32%, indicating that lesion pathology occurred in the same location in 32% of the patients. The probability map intersected with 19.9% of the CST, 19.2% of the IHST and 12.6% of the CTT. The maximum proportions of patients with lesions intersecting with these tracts were only 28.6% for the CST and IHST and 14.3% for the CTT.

We calculated the intersection between the motor tracts of interest and the lesion probability map to explore the relation between lesion load and fibre-specific damage (Fig. 4). While the probability map overlapped with 19.9% of the CST, 19.2% of the IHST and 12.6% of the CTT, the maximum proportions of patients with lesions intersecting with these tracts were only 28.6% for the CST and IHST and 14.3% for the CTT.

We observed a range of moderate correlations between conventional markers and fibre metrics (Supplementary Table 1), however, none survived FDR correction.

### Correlations with disability, motor performance and gait

**Figure 5:**
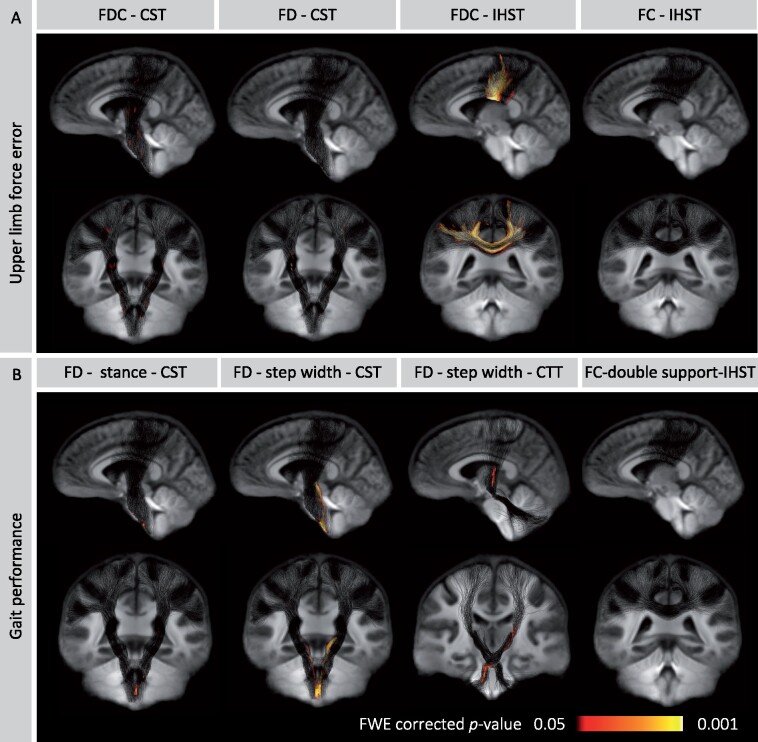
**Motor dysfunction and axonal damage.** **(A)** The associations between upper limb force error and fixel-metrics within the motor tracts. Greater force error during the upper limb visually guided force-matching task was associated with loss of fibre bundle density (FD) and fibre density and cross-section (FDC) in CST and reduced FDC and fibre bundle cross-section (FC) within the IHST. **(B)** Shorter stance was associated with reduced FD within the CST. Smaller step width was associated with reduced FD within the CST and CTT. Increased double support time was associated with reduced FC within the IHST. Areas of significance are coloured with the *p*-values [family-wise error (FWE) correction] and visualized on tracts using *p*-value.

Greater force error during upper limb force matching was associated with lower FDC in CST and IHST, lower FD within the CST and lower FC within the IHST (Fig. 5). Shorter stance was associated with lower FD within the CST. Smaller step width was associated with reduced FD in the CST and CTT. Increased double support time was associated with reduced FC within the IHST (Fig. 5). While the associations between axonal loss and functional measures appeared to be quite localized, uncorrected *p*-value maps (Supplementary Fig. 3) showed that the effect was widely distributed throughout the tracts, suggesting that family-wise error correction could lead to an underestimation of the anatomical extent of damage.

Fixel metrics, averaged across regions showing significant group differences, did not correlate with the EDSS in any tract.

## Discussion

Here we aimed to investigate and compare impairments in gait and motor control using advanced kinematic technologies, and axonal loss within key sensorimotor tracts of the brain in minimally disabled people with MS. The main findings of this study were: Minimally disabled MS patients (i) showed substantial loss of FC and FDC but minimal to no loss of FD along all motor tracts compared to HC and (ii) pathway-specific disruptions were associated with variation in upper limb force tracking performance and altered spatiotemporal patterns of gait.

### Extensive axonal loss along motor pathway in multiple sclerosis

As hypothesized, we observed axonal damage within all tracts of interest. The greatest effects were observed as loss of cross-section of fibre bundles, an indication of tract-specific atrophy. When we subdivided the CST into upper/lower limb and sensory/motor fibres, a larger proportion of S1 tracts compared to M1, and a larger proportion of lower compared to upper limb tracts were affected. We observed moderate correlations (not significant at an FDR corrected level) between CST FC and whole-brain lesion load, S1 atrophy and thalamic atrophy, but no correlation with cervical cord lesion load or cross-sectional area where most CST tracts terminate^29^ and lesions are commonly located.^30^ Consistent with recent reports,^31^ we observed relatively little primary lesion activity was present within CST, so axonal loss within these tracts could be attributable to spinal cord lesions or trans-synaptic degeneration due to lesions in second-order tracts. While we did not see a correlation with cervical cord lesion volume, these results must be interpreted with caution as we could only identify lesions within the rostral segments (C1–C4) due to a lack of dedicated spinal cord imaging consistently acquired during routine monitoring scans.

Significant axonal loss was observed within the IHST within medial subcortical areas as well as within the corpus collosum. Similar to the CST, when subdividing the IHST into motor and sensory tracts, a larger portion of the tracts connecting the two sensory cortices were damaged compared to M1 fibres. The corpus callosum is involved in control of unimanual movements by inhibiting interference of the other hemisphere and asynchronous bimanual movements by transcallosal passage of motor signals and sensory feedback.^32^ Structural damage within the corpus callosum observed here is congruent with findings of WM abnormalities reported in DTI literature.^5^^,^^33^^,^^34^

Less is known about the clinical effects of injury to the CTT in MS,^35^ compared to CST and IHST. Atrophy of regions at either end of the CTT tracts, the cerebellum and thalamus, is observed even at early stages of MS,^36^^,^^37^ and has been associated with clinical disability^38^^,^^39^ and tremor.^35^

While FC and to a lesser degree FDC changes were widespread in the tracts studied, FD reduction was only observed within a small part of the IHST. Further work is required to map the spatial and temporal patterns of loss of axonal density compared to tract atrophy across the disease course. At face value, one might expect that initially density is reduced due to axonal transection in lesions and diffuse axonal degeneration, and that subsequently the tract will atrophy depending on the rigidity of the extracellular matrix and the presence of glial proliferation and scarring. Consistent with this hypothesis, a previous study of patients with early stage MS shortly following acute optic neuritis showed preferential loss of FD and very little change in FC in the optic radiations.^17^ A more recent study examining FC in a sample of MS patients representing a broad spectrum of disability, showed substantial FC changes that were more pronounced in progressive than relapsing phenotype patients.^16^

### Axonal damage, visuomotor control and kinematics of gait

In MS patients, worse visuomotor hand control was associated with greater axonal damage within the CST and IHST. Microstructural damage to the corpus callosum has been observed from early on in the disease, even prior to the presence of macroscopic lesions^33^ and has been related to clinical disability worsening,^5^ dexterity impairment^6^ and disease progression.^40^ In this study, we found that the location of the WM damage in the IHST was mainly observed posteriorly, a region that is thought to be involved in complex somatosensory interhemispheric information transfer,^41^ with S1 tracts damaged in a greater extent than M1 interhemispheric tracts. Together with previous findings, our results suggest that the structural disruption within the corpus callosum has an effect on upper limb motor functioning, possibly driven by disruptions of somatosensory interhemispheric integration.

Changes in gait were associated with axonal damage within the CST and CTT. Reduced FD was associated with shorter stance and smaller step width. This contrasts with previous studies that reported prolonged stance time^42^ and wider step width in MS.^42^^,^^43^ These studies either included various MS phenotypes^42^ or patients using walking aids and or pyramidal and cerebellar FSS scores equal or above 2.^43^ In contrast, patients in this study were selected based on having minimal pyramidal and cerebellar dysfunction. Potentially, patients employ different strategies to maintain stability during walking at different disease stages. We also observed that loss of FC within IHST was associated with longer double support times. This finding is congruent with previous research showing prolonged double limb support^18^^,^^42^^,^^44^ related to clinical disability scores.^45^ Structural damage within the sensory and motor interhemispheric tracts potentially interferes with the transcallosal passage of motor signals and sensory feedback needed for bilateral coordination.^32^ Prolonged double support may be therefore be a compensatory mechanism to increase stability as balance control deterioration has been previously reported in MS.^46^

### Methodological considerations

In this study, we used ultra-high field diffusion MRI. While ultra-high field imaging has advantages in terms of higher signal-to-noise ratio and therefore the ability to image at higher spatial resolution, for diffusion imaging which utilizes spin-echo echoplanar imaging sequences, ultra-high field is associated with worse B0 and B1 field inhomogeneities leading to signal attenuation and distortions, particularly observed in the inferior temporal and orbitofrontal cortices. These limitations on the regions available for study with diffusion imaging at ultra-high field were not of great relevance to this study as we selected tracts of interest distant to B0 and B1 inhomogeneity effects, allowing us to take full advantage of the signal-to-noise ratio and resolution gains.

There are several additional methodological issues affecting the interpretation of our data. Firstly, the number of participants included was relatively small in comparison to large clinical studies. This reflects barriers to participation related to the amount of testing required (∼2 h visit for MRI and ∼2 h visit for comprehensive gait laboratory testing). However, more comprehensive phenotypic assessments of smaller yet more homogeneous sample of patients (EDSS < 4 and pyramidal FSS ≤ 2) can provide useful convergent evidence. Indeed, despite the relatively small sample size, our assessments were sensitive enough to identify axonal damage and motor impairments in minimally disabled patients. Such sensitivity is important for developing markers that could be deployed in smaller and short duration phase II clinical trials required to justify larger trials with clinical end-points. However, associations should be interpreted with caution and larger study sizes would be recommended in future studies to confirm findings. Whilst the gait markers used here would be difficult to implement in large-scale studies due to the requirement for 3D video tracking in a laboratory setting, these techniques have a high reproducibility. However, other techniques such as wearable sensors are easy to deploy and have reasonable reproducibility.^47^ Secondly, longitudinal studies will be required to evaluate the within-patient sensitivity of fixel-based markers of axonal loss, and to determine whether longitudinal change in axonal density and/or atrophy is associated with motor progression. Thirdly, we interpret loss of FD and cross-section as markers of axonal loss based on a theoretical understanding of the molecular diffusion processes in brain tissue and histopathological data regarding the specific pathologies evident in the MS brain. Other advanced diffusion models have been proposed as markers of axonal density (e.g. NODDI^48^), however, few of these can be analysed within a fixel-based analysis framework as they do not model individual fibre elements. Lastly, although we focused on three major motor tracts that are highly relevant to motor behaviours, there is potential for damage to other tracts to play a role in motor dysfunctions.

## Conclusions

In conclusion, significant gait and lower limb motor control impairments were detected in minimally disabled people with MS that correlated with axonal loss in major sensorimotor pathways of the brain. Given that axonal loss is irreversible, the combined use of advanced imaging and kinematic markers could be used to identify patients at risk of more severe motor impairments as they emerge for more aggressive therapeutic interventions.

## Supplementary material

Supplementary material is available at *Brain Communications* online.

## Supplementary Material

fcab032_Supplementary_Material

## Abbreviations

CST: cortico-spinal tracts
CTT: cerebello-thalamic tracts
DWI: diffusion-weighted imaging
DTI: diffusion tensor imaging
EDSS: Expanded Disability Status Scale
FC: fibre cross-section
FD: fibre density
FDC: fibre density and cross-section
FDR: false discovery rate
fMRI: functional MRI
FOD: fibre orientation distributions
GM: grey matter
IHST: interhemispheric primary sensorimotor tracts
MS: multiple sclerosis
WM: white matter
