# Supplementary material for: Axonal loss in major sensorimotor tracts is associated with impaired motor performance in minimally disabled multiple sclerosis patients
Source: Brain Commun. 2021 Mar 16;3(2):fcab032. doi: 10.1093/braincomms/fcab032 (PMC8244644; doi:10.1093/braincomms/fcab032)
Supplement: fcab032_Supplementary_Material [file fcab032_supplementary_material.pdf]

Supplementary material

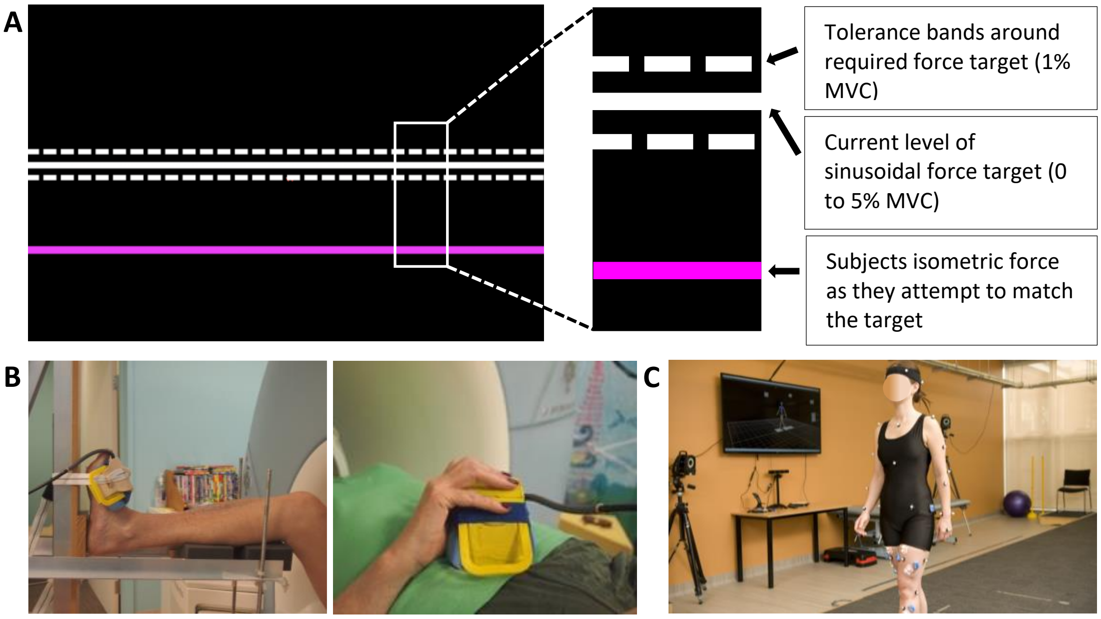

Supplementary Figure 1. Experimental set-ups for gait assessment and force tracking fMRI task. A) The visually-guided force-matching task presented to participants. The white line is the target force and in pink the participants' isometric force as they attempt to match the target force, which would slowly move up and down. Participants were asked to follow the target force as accurately as possibly by pulling their foot up and down or squeezing or releasing their hand. **B)** MR compatible rig and sphygmomanometer cuff positioned over the dorsum of foot, used to measure force production. **C)** Reflective markers were placed on specific body landmarks and a motion capture system was used to assess spatiotemporal patterns of gait. Patients were instructed to walk on a walkway at 1.4 ms^{-1}. Abbreviation: MVC = maximum voluntary contraction.

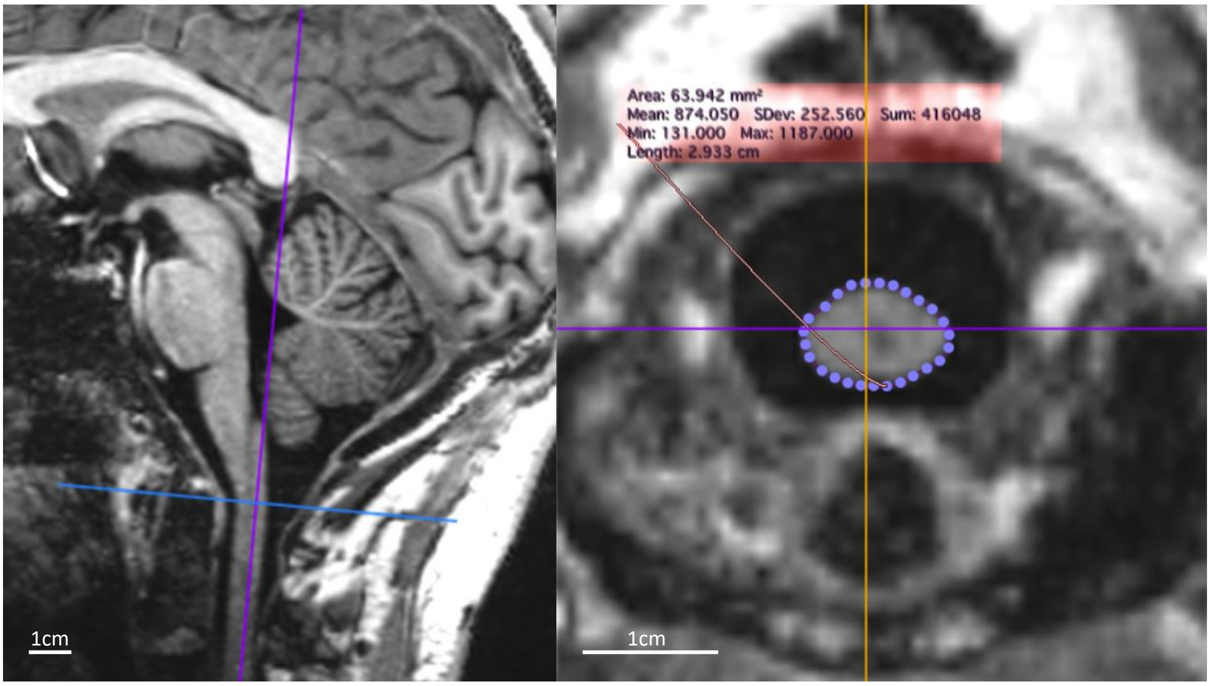

Supplementary Figure 2. Region of interest for spinal cord cross-sectional area measurement. The mid sagittal image was identified on MP2RAGE brain scans (left) and a transverse slice was identified at the rostral margin of the odontoid peg orthogonal to the caudally extending spinal cord (blue line). This slice was used to manually outline the spinal cord for all participants and the cross-sectional area was recorded.

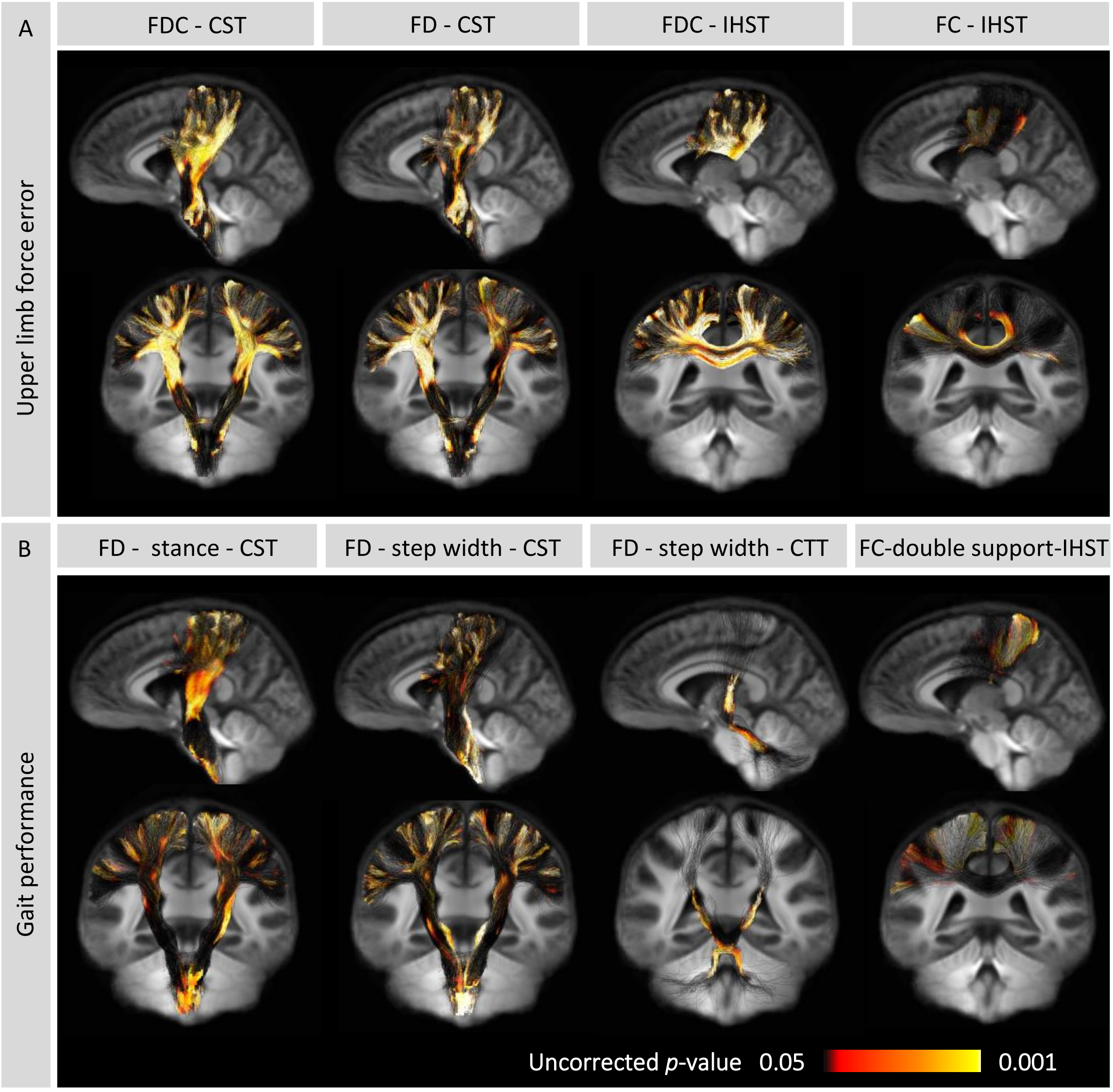

Supplementary Figure 3. Associations between motor dysfunction and axonal damage using uncorrected *p*-value. This image shows the same associations visualized in Figure 6, but with the uncorrected *p*-value plotted on the tractograms. As results of the relatively strict family wise error correction used in the analyses the location of the association between motor dysfunction and axonal damage seems to be quite moderate and localized. However, without correction associations were observed throughout the tracts, suggesting that loss of motor function is presumably not associated with localized damage but rather the entire tract might be involved. Abbreviations: FDC = fibre density and cross-section; CST = corticospinal tracts; FD = fibre bundle density; IHST = interhemispheric sensorimotor tracts; CTT = cerebello-thalamic tracts; FC = fibre cross-section.

Supplementary Table 1. Relationships fibre-specific measures and conventional MRI metrics. Linear regression were performed to assess the relationship between fibre-specific measures within areas of significant damage and volumetrics. Significant relations are highlighted in bold, but these associations do not survive FDR correction. All brain volumetrics were normalized for intracranial volume. Partial rank correlations were performed for ◊non-normally distributed data or *when residuals were non-normally disturbed. Abbreviations: S1 = primary somatosensory cortex; M1 = primary motor cortex; CGM = cortical grey matter; WM = white matter; CSA = cross-sectional area.

	**FC**			**FD**			**FDC**		
	p-value	$Beta_{std}$	p_{FDR}	*p*-value	$Beta_{std}$	p_{FDR}	*p*-value	$Beta_{std}$	p_{FDR}
**CST**									
Cerebellum, % ICV	0.333	0.228	0.642	0.125	0.293	0.422	0.074	0.361	0.375
Thalamus, % ICV	0.006	0.629	0.243	0.729	-0.071	0.937	0.192	0.280	0.536
S1, % ICV	**0.030**	**0.520**	**0.347**	0.169	-0.279	0.527	0.762	0.067	0.935
M1, % ICV	0.815	0.059	0.917	0.566	-0.119	0.917	0.816	-0.052	0.905
NCGM, % ICV	0.321	0.231	0.667	0.319	0.191	0.912	0.150	0.291	0.486
NWM, % ICV	**0.011**	**0.600**	**0.223**	0.328	-0.201	0.680	0.449	0.167	0.791
Brain lesions	**0.009**	**-0.585**	**0.243**	0.889	-0.028	0.664	0.100	-0.343	0.386
Spinal cord lesions◊	0.757	-0.064	0.943	0.493	-0.141	0.940	0.337	-0.196	0.635
Spinal cord CSA	0.813	0.058	0.928	0.888	0.028	0.922	0.720	0.077	0.941
IHST									
Cerebellum, % ICV	0.205	-0.300	0.536	0.050	0.372	0.312	0.210	0.261	0.532
Thalamus, % ICV	0.792*	0.056	0.930	0.081	0.349	0.365	**0.006***	**0.533**	**0.486**
S1, % ICV	0.578	0.141	0.918	0.710	0.078	0.943	0.330	0.216	0.652
M1, % ICV	0.364	-0.232	0.670	0.630	0.102	0.963	0.890	0.031	0.901
NCGM, % ICV	0.062	-0.429	0.359	**0.038**	**0.388**	**0.308**	0.285	0.221	0.624
NWM, % ICV	0.624	0.125	0.972	0.259	0.234	0.617	0.088	0.372	0.375
Brain lesions	0.729	0.086	0.923	**0.014**	**-0.467**	**0.227**	**0.045**	**-0.418**	**0.304**
Spinal cord lesions◊	0.873	-0.033	0.930	0.765	0.062	0.925	0.932	0.020	0.932
Spinal cord CSA	0.122	-0.373	0.430	0.276	0.223	0.621	0.671	0.092	0.937
CTT									
Cerebellum, % ICV	0.491	0.171	0.829	0.886	-0.039	0.932	0.704	0.121	0.950
Thalamus, % ICV	0.074	0.449	0.400	0.036	-0.564	0.324	0.368	-0.297	0.662
S1, % ICV	0.635	0.125	0.953	**0.035**	**-0.576**	**0.354**	0.170	-0.454	0.510
M1, % ICV	0.489	-0.183	0.843	0.830	-0.062	0.909	0.832	-0.073	0.899
NCGM, % ICV	0.651	-0.111	0.942	0.670	-0.114	0.952	0.691	-0.125	0.949
NWM, % ICV	0.092	0.432	0.373	**0.017**	**-0.645**	**0.230**	0.267	-0.371	0.618
Brain lesions	0.120	-0.379	0.442	0.190	0.356	0.550	0.767	0.097	0.914
Spinal cord lesions◊	0.511	-0.135	0.845	0.204	-0.257	0.551	0.217	-0.250	0.533
Spinal cord CSA	0.651	-0.116	0.959	**0.043**	**-0.537**	**0.317**	0.076	-0.562	0.362
